# Supporting laparoscopic general surgery training with digital technology: The United Kingdom and Ireland paradigm

**DOI:** 10.1186/s12893-021-01123-4

**Published:** 2021-03-08

**Authors:** Gemma Humm, Rhiannon L. Harries, Danail Stoyanov, Laurence B. Lovat

**Affiliations:** 1 Wellcome/EPSRC Centre for Interventional and Surgical Sciences, University College London, Charles Bell House, 43-45 Foley Street, London, W1W 7TY UK; 2 Division of Surgery and Interventional Science, University College London, London, UK; 3 Swansea Bay University Health Board, Swansea, Wales UK; 4 Department of Computer Science, University College London, London, UK

**Keywords:** Surgical training, Digital technology, Laparoscopic surgery, Simulation, Virtual reality mobile application, Artificial intelligence, Nontechnical skills, Telementoring

## Abstract

Surgical training in the UK and Ireland has faced challenges following the implementation of the European Working Time Directive and postgraduate training reform. The health services are undergoing a digital transformation; digital technology is remodelling the delivery of surgical care and surgical training. This review aims to critically evaluate key issues in laparoscopic general surgical training and the digital technology such as virtual and augmented reality, telementoring and automated workflow analysis and surgical skills assessment. We include pre-clinical, proof of concept research and commercial systems that are being developed to provide solutions. Digital surgical technology is evolving through interdisciplinary collaboration to provide widespread access to high-quality laparoscopic general surgery training and assessment. In the future this could lead to integrated, context-aware systems that support surgical teams in providing safer surgical care.

## Background

The health services in the United Kingdom and Ireland are undergoing a digital transformation [1, 2]. This has been mandated by the National Health Service (NHS) Long Term Plan and is supported by an additional £4.8 billion of Government investment in 2020–21 [3]. There is considerable support for the digitisation of the health services and the training of heath care professionals [4, 5]. Preparing the workforce for the changes in digital healthcare and training requires an infrastructure that facilitates multidisciplinary, collaborative work-placed learning with digital literacy incorporated into training [4]. Across surgical specialties, there is strong emphasis on surgical training and the development of innovate digital training in minimally invasive surgery [5]. Collaboration between surgical, engineering and computer science disciplines is crucial to ensuring the most critical issues will be addressed using innovative technology. Empowering surgeons to integrate technology into their roles could promote new concepts and the development and validation of new technologies, which can be facilitated by early adoption of technology into training, flexible surgical training and diverse Out of Program experiences [4, 5].

Surgical training in the UK and Ireland is overseen by the Joint Committee on Surgical Training (JCST) and the Surgical Royal Colleges for all surgical specialties. Training in General Surgery is a 6-year full time Specialty Training program which can be entered after passing the Intercollegiate Membership of the Royal College of Surgeons Exam and satisfactory completion of the Core Surgery (or equivalent) program. General Surgery trainees graduate from their program with a Certificate of Completion of Training (CCT) in General Surgery with a special interest. Table 1 summarises general surgical trainee operative experience requirements and index procedures that can be undertaken laparoscopically, training benchmarks indicate the stages of training that one would be expected to undertake cases related to special interest training [6–9]. Surgical training is undergoing a period of transition, with the introduction of an updated, competency-based curriculum (rather than time-based) which continues to support simulation training and should offer surgical trainees more flexibility in their training experiences [8–10].

**Table 1:** Summary of potential laparoscopic operative experience required through general surgery training [6–10] adapted from 2017 and 2021 core surgery and general surgery curricula

	ST3/Phase 2 Entry			ST4 benchmark		ST6/Phase 3 Entry benchmark		CCT	
		Level^c^	Number^d^	Level^c^	Number^d^	Level^c^	Number^d^	Level^c^	Number^d^
	Coordination of camera and instruments from a 2D display during surgical endoscopy	2	–	–	–	–	–	–	–
	Placement of laparoscopic ports	1	–	–	–	–	–	–	–
	Procedures logged c	–	> 20	–	–	–	–	–	–
Operative experience	Appendicectomy	2	–	3	–	3	70	4	80
	Inguinal Hernia	–	–	–	–	4	50	–	–
	Cholecystectomy^a^ Cholecystectomy^b^	– –	– –	2 –	– –	4 4	40 60	4 4	50 110
	Anterior resection (high)^a^	–	–	1	–	2	5	4	30
	Segmental colectomy^a^	–	–	2	–	3	20	4	50
	Major UGI procedures^b^ •Liver resection •Pancreatic resection •Oesophagectomy •Gastrectomy •Anti-reflux surgery	–	–	2	–	2	10	3	35

^a^Colorectal special interest trainees

^b^UGI special interest trainees who will concentrate on either oesophogastric or hepatopancreaticobilary procedures

^c^Competence levels 1—Has observed, 2a—Guidance required for most/all of the procedure (or part performed) 2b Guidance or intervention required for key steps only 3a—Procedure performed with minimal guidance or intervention (needed occasional help) 3b—Procedure performed competently without guidance or intervention but lacked fluency 4 a- Procedure performed fluently without guidance or intervention 4b—As 4a and was able to anticipate, avoid and/or deal with common problems/complications

^d^No specific proportion of laparoscopic cases specified

This review aims to identify issues facing surgical training and appraise the evidence of digital technologies that may support and facilitate laparoscopic general surgical training.

## Surgical training and laparoscopic general surgery

### Training opportunities

The implementation of European Working Time Directive (EWTD) and postgraduate reforms has reduced training time and operative experience. A comparison of 154 general surgical trainees’ logbooks was made in the first six months of implementation in August 2004 with the corresponding six months in 2003. Across the UK a 15.5% reduction in the overall logged cases was demonstrated [11]. Trainees reported an 11% and 18% reduction in coloproctology and upper gastrointestinal (UGI) surgery experience, respectively and an overall reduction index general surgery cases of 21% [11]. However, there is no literature detailing the specialist interest, or laparoscopic surgery logbook analysis. This is likely a reflection of the more generalised training received by trainees in the years before EWTD and the increase in special interest, centralisation and laparoscopic cases in the latter years. In another study, two surgeons’ operative were compared, demonstrating reductions in experience at “senior house officer” (SHO), approximately equivalent to core surgical training, level in both elective and emergency cases [12]. Surgeon A, reported working 100–120 h per week during a 12-month general surgery post in 1985–87, performed 35 elective inguinal hernia repairs, elective 22 cholecystectomies, 70 appendicectomies and 9 emergency laparotomies under supervision. Surgeon B, reported working approximately 48 h per week during 19 months general surgery experience in 2006–07, performed 6 elective inguinal hernia repairs, 1 elective cholecystectomy, 4 appendicectomies and 2 emergency laparotomies under supervision [12].

In Ireland surgical SHO operative experience data was collected prospectively over a 5-month period pre- and post-EWTD reduction in working hours [13]. Group 1 (August–December 2008) worked a median of 67 h per week (range 46–92), and Group 2 a median of 57 h per week (range 40–81). Total operative volume was reduced in Group 2 by 24% (p = 0.006). In Group 2, volume of cases assisted reduced by 26% (p = 0.005) and a reduction of 63% (p = 0.04), in intermediate cases (e.g., appendicectomy inguinal hernia repair) as primary operator but no reduction of minor cases (e.g. excision of skin lesion) as primary operator [13].

The reduction of surgical training opportunities has persisted. In 2017 a survey of 902 core surgical 0.3% of respondents’ posts adhered to all JCST generic quality indicators [9]. In general surgery, a 18.9% adherence was reported to the quality indicators; “Attend three supervised operating sessions (one of which should be an emergency session)” and “two supervised outpatient clinics each week” [14]. Failure to achieve quality indicators may put trainees who continue to higher surgical training in an inferior position to their peers by training inequality. The impact of this may not be seen until these trainees enter their consultant years.

One potential solution to the reduction in operating exposure is simulation training. However, this is not without its challenges. In a 2018 survey of 86.2% of respondents wanted improved access to simulation training [15].The survey results suggest there is regional variability in access to simulation training with 52.9% of respondents reported that there was access in their region. In one region, 27.3% of respondents reported no access to simulation training, in another region, 84% of respondents reported access at predetermined times. This survey identified that there may be inequitable access to simulation training, however, the nature of inequality is not explored and requires further research [15]. Delivering simulation training digitally could alleviate some inequality. Digital simulation could be delivered by devices that do not require dedicated laboratories and simulation teams to prepare and maintain or with portable devices that facilitate more flexible training in the hospital or at home.

### Virtual, augmented and mixed reality

Virtual reality (VR) is an artificial environment which is experienced through sensory stimuli provided by a computer in which one’s actions determine what happens in the environment [16]. VR in general surgery training can be in the form of in interactive digital platform accessed on a personal device [17] or a simulated laparoscopic operation with digital display and electronic sensors tracking instrument kinematics [18]. Different systems exist and can also include haptics [19] and immersive headsets [20].

A meta-analysis of randomized control trials (RCT) compared virtual reality (VR) simulation with either a box-trainer or no supplementary training in laparoscopic skills for junior trainees [21]. VR simulation significantly improved operative time and performance compared to no supplementary training or box-training [21]. Furthermore, structured surgical coaching was added to medical student training in VR laparoscopic cholecystectomy, comparing coaching plus instructional video with instructional video alone [22]. This RCT demonstrated the technical skills, assessed by blinded observers, using the Competency Assessment Tool (CAT) were consistently and significantly higher in the intervention group across multiple attempts with a significant reduction of errors and a plateau in the proficiency-gain curve of the intervention group [22].

Augmented and mixed reality describe systems whereby a virtual environment is overlayed and blended with the real environment [23], which can be viewed on immersive headsets. This technology has had limited use in simulating laparoscopic surgery, but is supporting the use of patient-specific anatomical models from preoperative imaging, to facilitate preoperative planning and training [24]. Operative guidance and navigation will require successful registration of images to patients, and is dependent on fixed anatomy and preferably with or within a bony frame [25]. As some abdominal organs are inherently mobile and move with pneumoperitoneum and ventilation, an environment which is not present during preoperative scanning, this technology may be less suited to laparoscopic abdominal surgery. Mixed reality and immersive headsets have been used during a case of robotic-assisted Transanal Total Mesorectal Excision of the rectum (TaTME). A trainee and assistant were able to view video feed from robotic (abdominal) camera and the endoscopic (rectal) camera in a single patient feasibility study where time worn was limited [26].

Another possible barrier to the use of this technology in laparoscopic surgery is that the operative field is visualised on a separate screen, and the axis is directed away from the patient. Using “see through” immersive headsets as an alternative means for the operating surgeon to view the endoscopic video has been used in cases of ureteroscopy [27, 28]. In the simulated setting the mixed reality headset facilitated the simultaneous viewing of endoscopic video, preoperative imaging and fluoroscopy and was found to reduce operative time and OSATS scores [28]. At the time of writing there were no published studies of a similar use of immersive headsets in laparoscopic abdominal/pelvic surgery. Augmented reality laparoscopic surgical navigation is likely to improve with systems whereby patient-specific anatomical images are overlayed onto intraoperative video and viewed on the laparoscopic screen. Such systems have been demonstrated in laparoscopic adrenalectomy [29] and laparoscopic liver resection [30]. At present the clinical application of this technology is still in its early stages and has the potential to benefit surgeon, patient and trainee in the future.

Digital simulation training has the potential to support surgical trainees in the development of clinical skills outside of the operating theatre. Access to more traditional forms of surgical simulation, such as dry- or wet-lab box trainer simulation training may not be possible for all trainees due to variations in regional training simulation resources [15]. A mobile, digital solution may help to mitigate this problem.

### Mobile application simulation

Mobile applications can provide an opportunity for surgical trainees to enhance their surgical cognitive skills outside the operating theatre, maximising their opportunities in the operating theatre. Mobile applications can provide high-fidelity simulation using computer generated graphics or surgical video. It is possible to enhance the fidelity of simulation using surgical video [31] and provide additional teaching information using graphical overlay to delineate anatomy [32] and additionally provide explanatory labels, annotations and multiple choice questions. New systems are under development validation, and are available for multiple surgical specialities [32, 33]. There are two platforms that are available for minimally invasive general surgery.

iLapp Surgery^TM^ is a subscription e-learning platform with modules for training in TaTME and laparoscopic hepatic resections [34]. This system includes a mobile application to support the use of the website. The TaTME modules includes cognitive training in the intraoperative steps with surgical video that can be displaced on the mobile device using Video in Picture. TaTME is a surgical technique that a surgical trainee in the UK and Ireland would be expected to have a level of understanding of, but would not be expected to have performed [6–8].This application may be of interest to trainees with a declared interest in colorectal surgery in their later stages of training, or those in post-CCT fellowship training. Trainees with a declared interest hepatobiliary surgery would be expected to have has exposure performing hepatic resections during their pre-CCT training, and may find this platform useful [6, 7]. At the time of writing there was no peer-reviewed studies to validate this platform as an educational tool.

Touch Surgery™(TS) [17], by Digital Surgery Ltd, is a free, downloadable mobile application which simulates surgery using an interactive, virtual environment on a personal device (phone or tablet). TS uses cognitive task analysis to teach and test knowledge and decision making in the process of surgical procedures. Multiple computer-generated simulations and surgical video simulations are available across all surgical specialties, including index cases, providing content that could be valuable for surgical trainees at all stages of training.

A study of in 51 medical students and 54 surgeons (33 junior trainee, 15 senior trainee, 5 consultants, 1 other) demonstrated face, content and construct validity in TS laparoscopic cholecystectomy modules [35]. In a randomised cross-over study of 52 medical students there was no significant transfer of cognitive skills from TS to a VR laparoscopic skills trainer. However, there was a significant improvement in TS modules scores following VR simulation training, transfer of technical skills was not demonstrated by either group [35].

In a further RCT of medical students attending a laparoscopic skills course, all received the same preparatory didactic teaching and dry-laboratory orientation prior to randomisation to receive either written information on the procedural steps of laparoscopic cholecystectomy or TS laparoscopic cholecystectomy modules [36]. Participants performed a laparoscopic cholecystectomy on a wet-laboratory porcine model, assessed by blinded video analysis using a score modified from a previous study [37]. Chidambaram et al. demonstrate there was an overall improvement in the cognitive performance in students randomised to the TS modules, compared to the control group, with higher overall mean performance score in the group randomised to TS compared to the control group, 41.9 ± 22.5 and 24.7 ± 19.6, respectively (p = 0.016). Analysis of the cognitive performance of subjects for the steps of laparoscopic cholecystectomy (initial exposure, initial dissection, cystic duct dissection, cystic artery dissection and gallbladder fossa dissection) were all higher in the group randomised to TS, but did not reach statistical significance [36].

### Mentored training

Mentored surgical training has been shown to support trainees at junior and senior levels in gaining surgical skills. Sixteen surgical trainees with limited laparoscopic experience performed a laparoscopic cholecystectomy. Eight consecutive trainees performed their first supervised laparoscopic cholecystectomy without additional coaching, then performed another case within two weeks. Trainers were instructed to provide verbal instruction if patient safety was compromised. The subsequent eight consecutive trainees performed their first supervised laparoscopic cholecystectomy followed by a sixty-minute structured coaching session with video playback. Their technical skills were subjectively assessed using video and a validated global assessment scale by blinded assessors. There were no significant differences between the two groups at initial assessment, however the group receiving structured feedback demonstrated significant improvement in the global assessment scale, economy of movement and operative time between their two cases [38].

Surgeons’ self-taught proficiency-gain curves for laparoscopic colorectal surgery was considered to be between 150 and 200 cases [39]. A structured, mentored National Training Program, (LAPCO™) was introduced to support UK surgeons to gain proficiency safely and efficiently in laparoscopic colorectal surgery [40]. Fourteen post-CCT trainees’ proficiency-gain curves demonstrated self-perceived Global Assessment Score (GAS) competence at 40 cases for mobilisation of the left colon, vascular pedicle division and anastomosis with comparable clinical outcomes to consultants. However, for more complex phases of operation (e.g. mobilisation of the splenic flexure and dissection of the mesorectum) self-perceived GAS competence was not achieved by 40 cases, nor during the study [41]. Risk-adjusted cumulative sum analysis for conversion to an open procedure demonstrated trainees took 24 cases to competently complete the case laparoscopically, under supervision. Of the 608 cases, 9.2% were converted to open, compared to a conversion rate of 10.6% reported in the National Bowel Cancer Audit [41]. The study reported an in-hospital mortality of 0.8% and overall complication rate of 16.9% and compared their result to meta-analysis data on in-hospital mortality (0.8%) and overall complications (18.2%) [41]. Mackenzie et al. argued colorectal trainees achieved satisfactory outcomes [41]. However, oncological outcomes were not analysed. This study demonstrates trainees with limited laparoscopic skills can be trained in laparoscopic colorectal surgery safely with mentored training. These studies hold some relevance to current colorectal trainees who have a better access to laparoscopic colorectal cases before CCT. It has since been shown that trainees who received mentored laparoscopic colorectal training achieved surgical outcomes comparable with consultants with no significant difference in short- and medium-term oncological outcomes [42]. Table 1 highlights JCST general surgical trainee operative experience requirements and index procedures that can be undertaken laparoscopically. Training benchmarks indicate the stages of training that one would be expected to undertake cases related to special interest training.

Not all surgical trainees have equitable access to mentoring and coaching. Enhancing secure multimedia communication and between trainees and trainers can facilitate learning through discussion and video case review. These interactions can support 1:1 coaching, group learning and peer to peer assessment and support using telementoring.

### Telementoring

AR telementoring in surgery can permit external communication with audio and visual input and telestration (graphical overlay on a live image). This can be established from a disparate site within the hospital or internationally [43, 44]. In a RCT of 19 medical students comparing AR telementoring and traditional mentoring to support dry-lab laparoscopic skills training demonstrated faster skill acquisition in laparoscopic suturing in the intervention group [45]. A study demonstrated the feasibility of telementoring in laparoscopic colorectal surgery and stated that whilst telementored cases took longer, patients had a reduced length of stay and a lymph node yield equivalent to standard cases. However, these results may be influenced by case selection [46]. AR telementoring and telestration technology has evolved, systems such as Proxime^TM^ [43, 47] are now commercially available and allows viewing of live and recorded intraoperative video, telestration and supporting digital data and interactions with other individuals or teams. This could be utilised by trainees and trainers, an integrated surgical command centre or experts in the field, and data could be securely stored for developing technical skill and team performance metrics [47].

## Technical skills

### Technical skill influences clinical outcomes

Surgical errors and poor technical skill in laparoscopic colorectal and UGI surgery are associated with an increased risk of postoperative mortality and morbidity [48–50]. Twenty bariatric surgeons from the United States of America (USA), each submitted a single representative laparoscopic gastric bypass video with clinical data for peer-peer assessment of technical skills. Edited videos were analysed by a minimum of ten peer-raters, and surgeons were allocated to quartiles. Higher surgical skill scores were consistently and significantly correlated with reduced risk-adjusted complication rates and postoperative mortality [50]. In a study of 61 laparoscopic gastrectomy cases unedited videos were analysed using Objective Structured Assessment of Technical Skills (OSATS). A global OSATS score of ≤ 29, the boundary of low performance, was shown to be an independent predictor of major short-term postoperative outcomes (odds ratio 6.49, 95% confidence interval 1.6–26.39) after adjusting for comorbidity and type of resection [48].

One hundred and seventy-five unedited laparoscopic TaTME videos were analysed using Observational Clinical Human Reliability Analysis (OCHRA) [49]. Curtis et al. argue inconsequential error, or ‘near misses’ are both prevalent and underreported with greater clinical significance. Patients with higher recorded rates of errors had more early postoperative complications. Cases with a significant intraoperative event demonstrated significantly greater number of ‘near misses’, compared to cases without significant intraoperative events [49].

### Assessing technical skills

The assessment of surgical technical skills in the UK and Ireland surgical training program is performed with an assessment of clinical skills, as part of a Procedure-Based Assessment (PBA). Trainees are observed by trainers and assigned a criterion-referenced global rating scale from 1 to 4, indicative of competency, where 4 is independent (Table 1). The level of supervision required decreased with time and increased experience, reflected in the global rating scale [51]. PBAs have been shown to be a reliable method of procedure-specific performance in a prospective cohort of motivated trainees and trainers [52]. However, the correct and timely usage of work based assessments is variable, with discrepancies in the perception of delivered and received feedback [53]. Table 1 details the indicative numbers and the global rating level required of trainees at training benchmarks.

In technical skills research total operative time has been used as a surrogate marker of performance. Several tools for the objective assessment of technical skills have been described. Global assessment of surgical skills scores include: GAS [40], PBA [54], OSATS (originally validated for open surgery) [55], Colorectal OSATS [56, 57] Global Operative Assessment of Laparoscopic Skills (GOALS) for laparoscopic skills [58, 59] and Structured Assessment of Laparoscopic Assistant Skills (SALAS) for laparoscopic camera navigation [60]. Each assess skills over a number of domains, on 5-point score. The identification and classification of surgical error includes an unnamed score specific to laparoscopic cholecystectomy [37] the Generic Error Rating Tool (GERT) [61] and OCHRA which can be applied to any operation [49, 62, 63]. Individually these tools have been validated for use in different clinical [63] and educational settings [66], including direct observation and delayed video analysis. Tools for delayed video analysis can be difficult and time consuming to use and are rarely used outside of research or proctored courses, resulting in underutilisation and the valuable insights missed. Recording and reviewing cases for the purposes of operative documentation and education could be of benefit of surgeons, surgical trainees and hospitals [67, 68]: a secure, digital solution to automatically store surgical video, with the potential to analyse intraoperative phase times and technical skills would be advantageous by improving access, security and reducing annotation workload [69].

### Artificial intelligence and laparoscopic video analysis

Laparoscopic surgery is dependent on a live video feed, which is infrequently recorded as standard of care. Surgical video is used for education and training [68] and inclusion of surgical video improves the accuracy of intraoperative documentation in laparoscopic colorectal surgery [67]. If appropriately captured, surgical video data could support the development of digital technology. Online surgical communities host video libraries for education and training and offer subscribers the opportunity to view peer-reviewed, edited and narrated cases [70] whilst others offer secure platforms to store and automatically analyse intraoperative times and phases for self and unit evaluation [71] and provide peer-peer assessment of technical skill [72].

Artificial intelligence (AI) networks can be used to extract and analyse visuospatial data in laparoscopic video to classify images in sequence. This technology can identify intraoperative phases and transitions and identify and track laparoscopic tools, which allows interpretation of variability between cases and surgeons. Understanding the surgical workflow allows surgical teams to reveal variability that may have otherwise gone undetected. The surgical workflow can be standardised within or across units or individualised to reflect challenging cases. Supervised learning techniques, whereby AI networks are trained using expert annotation, have been demonstrated to predict the intraoperative phase in laparoscopic cholecystectomy video with an accuracy of 88.9% ± 7.5% [73], sleeve gastrectomy; 82% ± 4% videos [74] and laparoscopic left sided colorectal resections; 81% (no standard deviation reported) [75]. The recognition and tracking of laparoscopic instruments has been achieved [73, 75] and analysing instrument kinematics can enable interpretation of surgical skill [76]. This may support the future development of software to assess surgical skills and identify surgical errors.

The ability of computer systems to ‘understand’ what is happening in the operating theatre is also known as a ‘context-aware’ system. This data and analysis can be harnessed to drive digital displays of clinical information such as pre-operative imaging, instruments, learning points and intraoperative steps to provide the surgical team with the information required to both prepare for, to progress and complete the case [77]. Such systems have the potential to improve theatre efficiency and surgical training.

## Nontechnical skills

### Team familiarity

A high turnover of operating theatre staff may contributed unfamiliarity in the operating theatre, In England operating theatre staff increased by 41% between 2009 and 2018 [78]. Graphical data suggests between October 2018 and March 2019 there were approximately 20,000 and 75,000 advertised full time equivalent vacancies in “medical and dental” and “nursing and midwifery registered” posts, respectively, which could represent a conservative estimate in NHS England, due to the variable definition of vacancy [79]. Staff shortages and agency staff may result in a high turnover of staff in the operating theatre, with many staff working in an unfamiliar environment. Unfamiliarity with the procedure, policy, team and environment [80] was cited as a contributing factor to ‘Never Events’, which including retained foreign objects, wrong site surgery and wrong implant surgery [80]. Whilst direct causality cannot be implied where a number of systemic failures are present, improving staff ‘familiarity’ could improve patient safety. Improving the cognitive skills and knowledge of the surgical workflow by accessing digital simulations in advance [32, 35, 36] and utilise AI compatible digital intraoperative workflow could be beneficial for all members of the surgical team in an unfamiliar case and/or environment.

### Assessing nontechnical skills

Lower nontechnical skills scores are associated with observed miscommunications and interruptions across multiple surgical specialties [81]. An observational study in the USA documented observed team behaviours and 30-day post-operative outcomes [82]. Operations with lower frequencies of observed behaviour across the domains “briefing”, “information sharing”, “inquiry” and “vigilance” over the induction, intraoperative and handoff phases of the operation, were associated with postoperative complications and death, when corrected for comorbidity [82].

Analysis of the surgical team’s nontechnical skills was undertaken using synchronized laparoscopic, operating theatre and audio recordings. These recordings were analysed by an expert team to identify safety threats across the domains, external environment, physical environment, organization, tools and technology, tasks and person. One surgeon and their team identified a total of 499 safety threats over 19 cases (39.8 h) of complex laparoscopic UGI surgery (mean per case 26 ± SD18) and a total of 584 (mean per case 31 ± SD19) resilience supports [83]. Unnecessary conversation during the execution of clinical tasks was a considerable safety threat, which was observed 30 times over 13 cases. Resilience supports included skills coaching, proactive delegation of tasks and consistent execution of the surgical time out [83]. Whilst technical skill and intraoperative errors are documented, a causal relationship between skill and error rate cannot be assumed. This study suggests many errors or ‘near misses’ were mitigated by nontechnical skills, such as skills coaching, verbalisation of completed tasks and proactive delegation [83]. Definitions of the individual safety threats and resilience supports were not provided. In a randomised control trial of 23 surgical residents comparing standard resident training and standard resident training plus a 2 month nontechnical skills curriculum with didactic and simulation training nontechnical skills were significantly improved in the intervention group as assessed by simulated crisis scenarios [84]. By improving surgeons’ nontechnical skills, it could be possible to improve clinical outcomes and improve patient safety.

### Team training

Team training has been shown to reduce operating theatre delays, promote communication and debriefing within the surgical team in a prospective study with a 24 month follow-up period [85]. Simulated operating theatre team training could benefit trainees in the surgical team, a systematic review which included surgical, anaesthetics and emergency medicine trainees team training in simulated crisis management skills demonstrated that trainees’ knowledge and nontechnical skills in the simulated environment improved with simulated team training [86].

There is evidence to support diversifying simulated individual and training for both clinical and nontechnical skills. Black box recording systems as described by Kolodzey et al. could provide an integrated digital solution for intraoperative documentation, morbidity and mortality review, supporting positive team interactions and team training.

## Discussion

Efficient training and the assessment of technical and nontechnical skills are key components of surgical training and contribute to a successful procedure. Increasing exposure to video libraries and structured, mentored training at individual and team levels will likely improve the cognitive, technical and nontechnical skills required to perform laparoscopic surgery. It is possible these skills can be transferred to other professional and clinical situations [22, 35, 36, 40, 42, 68, 87].

**Fig. 1:**
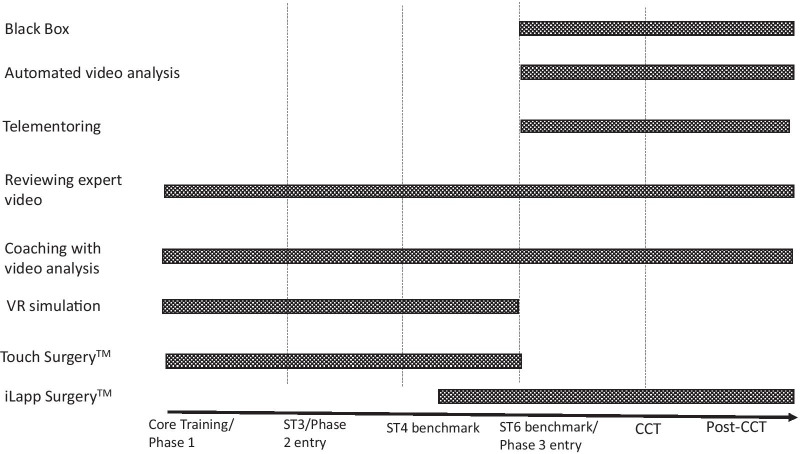
A suggested timeline for digital support in general surgical training

Enhancing surgical experience in early training, could have a positive effect on the recruitment and retention of doctors into higher surgical training and increase the rate of skill acquisition in senior trainees [14]. Training issues and potential digital solutions are summarised in Table 2 and a surgical training timeline is proposed for using these digital solutions in Fig. 1.

**Table 2:** Summary of key studies demonstrating potential digital solutions to training issues

Training issue	Potential digital solution	Key studies	Study design	Learner groups	Conclusions
Formative experience	Touch Surgery^TM^	Kowalewski et al. (2017) [35]	Randomised cross over study	Medical students Junior trainee Senior trainee	Face, content and construct validity of Touch Surgery^TM^ Cognitive skills transfer more successful with VR simulation Valuable training experience with Touch Surgery^TM^
	Touch Surgery^TM^	Chidambaram et al. (2019) [36]	RCT	Medical students	Superior cognitive performance scores in Touch Surgery^TM^ group compared to control
Acquisition of basic laparoscopic skills	VR Simulation	Nagendran et al. (2013 [21]	Systematic review and meta-analysis of RCTs	Surgical trainees	VR training improves operative performance compared to box-trainer or no supplementary training
Operative experience	Video-based education	Ahmet et al. (2018) [68]	Systematic review of RCTs	Medical students Surgical trainees	Video-based education associated with higher performance score and trainee satisfaction
Constructive feedback	Coaching with video analysis	Grantcharov et al. (2007) [38]	Interventional study	2 surgical trainees	Constructive coaching with video analysis improved global assessment score
	Coaching with VR Simulation	Cole et al. (2014) [22]	RCT	Junior trainees	Reduction in errors and improvement of CAT over time with additional coaching
Assessment of skill	Peer review of video	Birkmeyer et al.(2013) [50]	Observational/feasibility	Surgeons	Poor technical skills associated with postoperative complication Peer assessment of video successful
	Automated video assessment	Twinanda et al. (2017) [73]	Experimental	Surgeons	Accurate phase prediction and instrument recognition in laparoscopic cholecystectomy
	Automated video assessment	Hashimoto et al. (2019) [74]	Experimental	Surgeons	Accurate phase prediction and instrument recognition in laparoscopic sleeve gastrectomy video
	Automated video assessment	Jin et al. (2018) [76]	Experimental	Surgeons	Instrument tracking suitable for inferring laparoscopic skills in laparoscopic cholecystectomy video
	Automated video assessment	Kitaguchi et al. (2020) [75]	Experimental	Surgeons	Accurate phase prediction and instrument recognition in laparoscopic left sided-colorectal resection video
	Automated live video assessment	Winter Beaty et al. (2019) [52]	Experimental	Surgeons	Accurate phase prediction and in laparoscopic sleeve gastrectomy live video
Supporting independent operating	AR and Tele-surgery	Greenfield et al. (2018) [43]	Case Study	Surgeons	Successful international audio-visual communication with interactive graphical overlay
Surgical skill acquisition	AR telemontoring and simulated skills	Vera et al. (2014) [45]	RCT	Medical students	Faster skill acquisition in simulation with AR telementoring mentoring
Surgical teamwork	Black box analysis	Kolodzey et al.(2019) [83]	Observational	Single surgeon	Surgical “near misses’ prevented by non-technical skills
	Tele-surgery	Lin et al. (2020) (44)	Feasibility	Orthopaedic surgeons	Live streaming and tele-mentoring during arthroscopy

*AR* augmented reality, *VR* virtual reality, *CAT* Competency Assessment Tool

The uptake of laparoscopic colorectal surgery has increased and surgical trainees are likely to achieve a greater ratio of laparoscopic to open cases than previously [88]. However, specialist laparoscopic training in the UK and Ireland can be dependent on post-CCT fellowships. Many fellowships will follow the proctored model advocated by LAPCO^TM^, and may be taken at “home” or internationally [89]. It is possible that digitally connecting fellows and their trainers with telementoring could enrich the training and mentoring experience. Additionally, international collaboration could foster the consensus and standardization in training. A parallel can be drawn with robotic training. In the UK and Ireland there is currently no agreed or standardized robotic training program for any specialty [90]. Urology, as early adopters of robotic surgery, are an example. There is significant emphasis on pre-clinical simulation training, including model-specific VR simulation and curricula [91] and mentored training [92]. Robotic surgery additionally facilitates easy recording of intraoperative video and the publication of video datasets [93], which has supported the development of AI networks to recognise fine granularity movement of robotic instruments for surgical skills assessment [94]. In the UK and Ireland robotic UGI and colorectal surgery gains popularity, and is successfully being implemented in appropriate centres following mentored training [95, 96].

Studies that examine the application of surgical skills assessment scores to laparoscopic videos are often small, single centre observational studies with short follow up and simple mortality outcomes. Whilst these outcomes are of use, limiting outcome data to these parameters does not reflect other important outcomes, such as oncological outcomes or staff and patient acceptability. Neither is it possible to understand the relationship between specific errors or events to particular complications or outcomes [48–50]. Studies also show substantial heterogeneity in design and are likely subject to publication bias [64, 97].

The benefits of developing a digital system include democratising access to training and educational material and efficient and procedural support for surgical teams which can overall improve outcomes and robust, accessible and transparent documentation of intraoperative events. This technology could give further insights into predictions in postoperative morbidity to improve the delivery of care. Digitising a methodology for the assessment of laparoscopic technical skills in everyday practice and delivering structured feedback could improve technical skills. This may also improve the perception of delivered and received feedback, which could improve trainee-trainer relationships. Formalising additional opportunities for technical skills to be constructively assessed and recorded could empower surgeons to record and reflect upon their own progress. This could prompt a surgeon or unit to engage in further training to ensure best practice is achieved and be useful in appraisal and revalidation.

As these systems evolve care should be taken to ensure patient safety is always paramount. Early technology which infers the surgical process may lead to decision support devices, which may be met with trepidation by patients and clinicians alike. It is crucial clinician autonomy remains in the operating theatre. In the future technology that documents surgical team performance may become of increasing importance in cases of litigation and to insurance companies. In our opinion, technology should be used to drive standards higher across the specialty instead of creating new health inequalities.

For these aspirations to be actualized more data is required. Hospitals should consider recording all laparoscopic cases as standard of care, to improve documentation and generate video datasets that can be accessed and analysed retrospectively [67], increasing data accessibility and AI network training [73, 98]. Some hospitals may have difficulty securely storing video data for operative documentation. This is likely to improve as hospitals transition to paperless records.

This review has focussed on laparoscopic surgical training in the UK and Ireland and applied surgical training research and technological developments in Western countries which share similar healthcare systems. It is possible that other healthcare systems may lack some of the required equipment and infrastructure to implement and develop systems applicable to their own healthcare setting. However, the delivery of educational material via personal, portable devices continues to be a viable option as the use of smart phones in developing economies increases [99, 100].

## Conclusion

As digital technology continues to develop and support the training of surgical teams we can anticipate a more streamlined and efficient training system that supports the development of cognitive, technical and nontechnical skills simultaneously. Standardising and automating assessments can facilitate trainee progression, maximise the delivery of surgical care and improve outcomes for our patients.

Digital strategies appear to benefit surgeons, surgical teams and patients alike. As these tools develop and are enriched by the growing data available there is the possibility to provide widespread access to high quality training materials and assessment. In the future this could lead to standardised, regulated systems that can support surgical teams in providing safer surgical care.

## Data Availability

Not applicable.

## Abbreviations

AI: Artificial intelligence
AR: Augmented reality
CAT: Competency Assessment Tool
COSATS: Colorectal Objective Structured Assessment of Technical Skills
CCT: Certificate of completion of training
EWTD: European Working Time Directive
GAS: Global Assessment Score
GERT: Generic Error Rating Tool
GOALS: Global Operative Assessment of Laparoscopic Skills
JCST: Joint Committee on Surgical Training
NHS: National Health Service
OCHRA: Observational Clinical Human Reliability Assessment
OSATS: Objective Structured Assessment of Technical Skills
PBA: Procedure Based Assessment
RCT: Randomized Control Trial
SALAS: Structured Assessment of Laparoscopic Assistant Skills
SHO: Senior House Officer
TaTME: Transanal total mesorectal excision of the rectum
TS: Touch surgery™
UGI: Upper gastrointestinal
UK: United Kingdom
USA: United States of America
VR: Virtual reality
